# Laparoscopic vs percutaneous hysterectomy in obese patients: a prospective evaluation

**Published:** 2020-03-27

**Authors:** E Perrone, F Fanfani, C Rossitto, S Cianci, A Fagotti, S Restaino, C Fedele, G Scambia, S Gueli Alletti

**Affiliations:** Division of Gynecologic Oncology, Fondazione Policlinico Universitario A. Gemelli IRCCS, Rome, Italy;; Università Cattolica del Sacro Cuore, Istituto di Clinica Ostetrica e Ginecologia, Rome, Italy

**Keywords:** Minimally invasive surgery, percutaneous approach, new technology, hysterectomy, gynecological surgery, personalized surgical treatment

## Abstract

**Background:**

Treatment of obese female patients represents a real challenge. Indeed, obesity among women has reached epidemic levels not only elevating the cardiovascular and endocrinological risks, but also increasing the incidence of various gynecological pathologies (e.g. endometrial cancer and hyperplasia, uterine fibroids, genital prolapse) which commonly require hysterectomy as a surgical solution. In the last decade, minimally invasive surgery has emerged as an approach reducing the invasiveness of the standard laparoscopic surgical procedures while maintaining efficacy and feasibility. As such, in this study we aimed to evaluate the feasibility of percutaneous hysterectomy (PSS-H) approach in obese patients by reporting the first prospective comparison between the PSS-H to laparoscopic hysterectomy (LPS-H).

**Methods:**

In this multicentric comparative prospective study, 45 patients affected by benign and malignant gynecological conditions were considered eligible for minimally invasive surgery (MIS). Fifteen patients received PSS-H and 30 LPS-H. All patients enrolled received a total hysterectomy ± bilateral salpingo-oophorectomy, with or without lymph nodal staging.

**Results:**

No statistically significant differences were noted in operative time and estimated blood loss between the two groups. Four patients in PSS-H group and 3 in LPS-H group received lymph node staging. A multifunctional energy device was used in all PSS-H and 73.3% of LPS-H procedures (p=0.038). There were no conversions to laparotomy in either group and similarly there were no conversions to conventional laparoscopy in the PSS-H group. In the LPS-H group, there was one (3.3%) case of major bleeding( ≥ 500 mls). We recorded one vaginal cuff bleeding in PSS-H, whereas for LPS-H we reported 4 (13.3%) 30-days complications (p=0.651). No differences in visual analogue scale (VAS) score were recorded. A significant disparity was noted in cosmetic outcome at discharge (p=0.001), but not after 30 days.

**Conclusion:**

We demonstrated for the first time, in a prospective comparison between PSS and LPS approaches, that PSS-H may represent a valid alternative to performing total hysterectomy in obese patients.

## Introduction

Worldwide, obesity has reached epidemic proportions amongst women, increasing from 6.4% in 1975 to 14.9% in 2014 (NCD Risk Factor Collaboration, 2016). The World Health Organization (WHO) defines a body mass index (BMI) under 18.5 kg/ m2 underweight, a BMI between 18.5 and 24.9 kg/m2 normal, a BMI between 25 and 29.9 kg/ m2 as overweight, and a BMI ≥ 30 kg/m2 obese (WHO, 2000). Recent evidence reported an obesity prevalence of 40.4% among women and 35.0% among men. In particular, the prevalence of class 3 obesity (BMI ≥40), is significantly higher in women than men, 9.9% vs 5.5% in the United States (Flegal et al., 2016). These data indicate that female obesity is a real challenge for clinicians because, besides the elevated cardiovascular and endocrinological risks, it is known that obesity increases the incidence of a number of gynecological pathologies, such as endometrial cancer and hyperplasia, uterine fibroids, associated bleeding disorders and genital prolapse, commonly requiring hysterectomy as a surgical solution (Morgan-Ortiz et al., 2013).

For the patients with a surgical indication, hysterectomy is the most accessible and frequent surgical procedure (Wright et al., 2013) and the use of minimally invasive surgery (MIS) has become the gold standard to perform this surgical procedure (Turner et al., 2013). Several studies have confirmed that laparoscopic hysterectomy (LPS-H) is superior to laparotomic hysterectomy, reaching equal medical effectiveness in reduction of hospitalization, post-operative pain, and the rate of complications, leading to a consistently better outcome in the patient’s quality of life (Kluivers et al., 2008; Janda et al., 2010; Fortin et al., 2019).

Despite obesity commonly being defined as an independent risk factor, it increases the difficulty of performing LPS-H, being associated with longer operative times and higher complication rates (Shah et al., 2015). Nevertheless, LPS-H is still considered to be superior to open hysterectomy (Osler et al., 2011). However, the minimally invasive approach is generally considered technically more complex, due to the difficulties in accessing the abdominal cavity in the presence of increased thickness of the abdominal wall and visualization of the surgical field (Eltabbakh et al., 1999).

In the last decade, many technological improvements occurred in MIS. Development of new techniques improved standard laparoscopic surgery further, reducing the invasiveness whilst maintaining the efficacy and feasibility of the standard technique. 3mm laparoscopy (M-LPS) and single-port surgery (SP) are such examples (Moulton et al., 2017, Beguinot et al., 2020). Percuvance TM (Percutaneous Surgical System (PSS), Teleflex Inc., USA) is the lastest innovative ultra-MIS technique. PSS is characterized by percutaneous instruments, with less than 3mm diameter, percutaneously introduced into the abdomen without the need of trocars. Differently from M-LPS, this system is equipped with a 5 mm operative tip which is hooked on the 2.9 mm shaft through a 5 mm suprapubic trocar. With these characteristics, PSS simulates the instruments and triangulation setting of standard laparoscopy, with a further reduction in invasiveness (Rossitto et al., 2016, Rossitto et al., 2017). Several studies have reported the feasibility of this new tool, both in benign and early malignant gynecological conditions demonstrating its comparability with other minimally and ultra-minimally invasive approaches (Rossitto et al., 2016, 2017; Gueli Alletti et al., 2017, Gueli Alletti et al., 2019). Nevertheless, to our knowledge, the feasibility and outcome of PSS in obese patients is lacking in the literature and focussed studies are required to assess limits and potential advantages of this new tool in these patients.

Consistent with this view, in this study, we report the first prospective comparison between the percutaneous hysterectomies (PSS-H) to LPS-H in a series of obese patients.

## Materials and methods

### 

In this study we prospectively compared 15 consecutive percutaneous hysterectomies (PSS- H, study group) with a cohort of 30 laparoscopic hysterectomies (LPS-H, controls). All patients (N=45) were obese (BMI≥30) and were subjected to elective hysterectomy. Patient selection was conducted through gynecologic examination plus transvaginal ultrasound, preoperative biopsy and further magnetic resonance imaging (MRI) or computerised tomography (CT) scan when indicated. Patients diagnoses were benign (fibroids, adenomyosis, and endometriosis), pre-neoplastic (CIN2/3, typical and atypical hyperplasia) or low/intermediate risk endometrial cancer, FIGO stage IA G1-G2, IB G1-G2, IA G3, eligible for both percutaneous or laparoscopic hysterectomy (Table I). Patients with preoperative suspicious cervical or lymph nodal involvement or with an American Society of Anesthesiologist Score > III were excluded. Enrolled patients received pre- operative counselling about the surgical techniques and signed written informed consent, as approved by the Institutional Review Board. All surgical procedures were performed, from August 2015 to November 2017, by 5 surgeons fully expert in MIS in the Department of Woman and Child Health and Public Health, Fondazione Policlinico Universitario A. Gemelli IRCCS in Rome, in Pineta Grande Hospital, Castel Volturno, Italy and in Division of Gynecology, San Carlo di Nancy Hospital, Rome, Italy.

**Table I:** — Patients characteristics according to surgical approach.

BASELINE CHARACTERISTICS:				
Characteristics		PSS	LPS	P value
N of patients		N= 15	N= 30	
Age (year), median (range)		60 (47-80)	55 (31-79)	0.360
BMI (kg/m^2^), median (range)		30.4 (30-37.9)	31.8 (30-41)	0.022
Previous abdomino-pelvic surgery, n (%)		10 (66.7)	19 (63.3)	0.826
Indications for hysterectomy, n (%)				
	Fibroids/Adenomyosis	5 (33.3)	12 (40.0)	0.667
	Endometrial hyperplasia	0 (0.0)	6 (20.0)	0.073
	Endometrial cancer	10 (66.7)	11 (36.7)	0.057
	BRCA or familial history	0 (0.0)	1 (3.3)	0.659

Results are presented as n (%) or median (range). PSS: Percutaneous Surgical System; LPS: Laparoscopy. BMI: body mass index.

The total extrafascial hysterectomies with or without bilateral salpingo-oophorectomies were conducted, in both approaches, as reported previously (Gueli Alletti et al., 2019). When clinically required, according to National Comprenhensive Cancer Network (NCCN) guidelines, the lymph nodal assessment was performed in endometrial cancer cases. According to the technological evolution in endometrial cancer staging in recent years, the lymph node staging consisted of a systematic pelvic lymphadenectomy or sentinel lymph nodes mapping.

**Figure 1:**
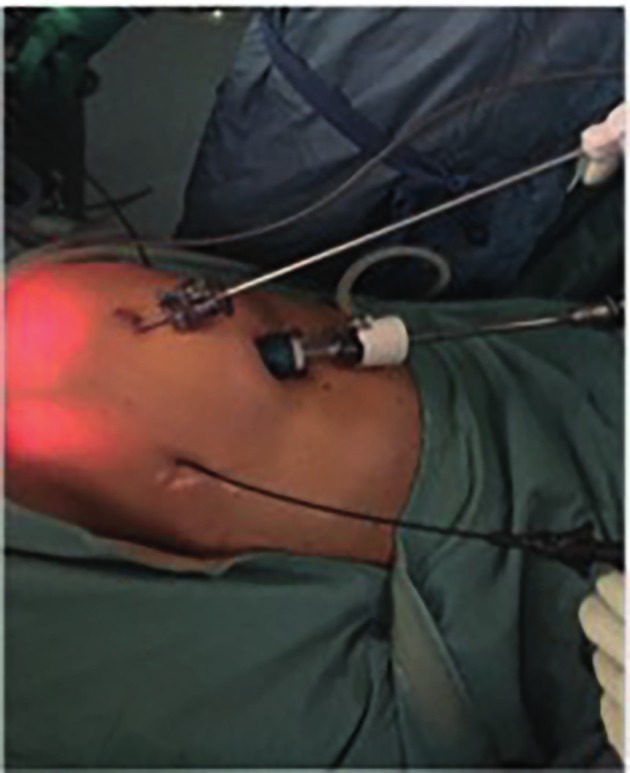
External view of the percutaneous system setting during a percuteneous hysterectomy.

**Figure 2:**
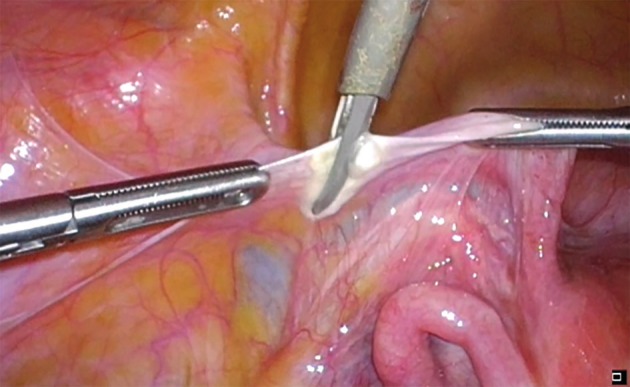
Internal view of the percutaneous instruments together with the multifunctional 5 mm energy device.

The pneumoperitoneum (12 mmHg) was achieved, through the open-laparoscopy technique, using a Hasson trocar for both approaches. We used a 0° HD telescope (ENDOEYE, Olympus Winter & Ibe, Hamburg, Germany). After that, in the PSS-H setting, we inserted one suprapubic standard 5-mm port and two PSS instruments were used as side graspers for both surgeon and first assistant. The Percuvance^TM^ installation was conducted percutaneously inserting the needle tip of the instruments. Then, using the 5 mm suprapubic port, the needle was replaced by the functional tip (gripper or alligator grasper tip) for both percutaneous instruments. Using the 5 mm port, we used a suction/irrigation device, monopolar hook, 5 mm endoclip, and multifunctional instrument (Thunderbeat, Olympus Winter & IBE GMBH, Hamburg, Germany) (Figures 1 and 2). In the LPS-H group, we used three 5 mm ports: one suprapubic port, one in the left lower quadrant and one in the right lower quadrant. The multifunctional instrument was used in both approaches but was the only energy device in PSS approach due to lack of bipolar energy in percutaneous instruments.

Pre- and postoperative data were prospectively collected in an electronic database. The total operative time (OT, calculated skin to skin), vaginal cuff closure time and other intra- and postoperative data, are presented in Table II. Any injury to the bowel, bladder, ureters, nerves or blood vessels or an estimated blood loss (EBL) ≥ 500 ml were defined as intra-operative complications. Patients were discharged when they were afebrile and in good clinical condition. Thirty days after surgery we recorded post-operative complications, defined as fever > 38°C, excluding the first day after surgery, vaginal dehiscence, bleeding or hematoma, bowel occlusion, post-operative infection, and need for a second surgical procedure.

**Table II:** — Perioperative outcomes according to surgical approach.

PERIOPERATIVE OUTCOMES:				
Characteristics		PSS	LPS	P value
N of patients		N= 15	N= 30	
Surgical procedure:				
	Bilateral Oophorectomy, n (%)	15 (100.0)	28 (93.3)	0.545
	Lymph nodal staging, n (%)	4 (26.7)	3 (10.9)	0.199
Multifunctional instrument, n (%)		15 (100.0)	22 (73.3)	0.038
Operative time (min), median (range)		90 (45-180)	94 (35-210)	0.587
Vaginal suture				0.034
	Laparoscopy, n (%)	10 (66.7)	10 (33.3)	
	Vaginal, n (%)	5 (33.3)	20 (66.7)	
Manipulator, n (%)		12 (80.0)	21 (70.0)	0.722
EBL (ml), median (range)		50 (50-200)	100 (0-500)	0.252
Uterus weight (gr), median (range)^a^		145 (60-350)	180 (45-1800)	0.443
Drain insertion, n (%)		8 (53.3)	19 (63.3)	0.519
LPT conversion		0 (0.0)	0 (0.0)	1.000
Intraoperative complications, n (%)		0 (0.0)	1 (3.3)	1.000
Postoperative complications, n (%)		1(6.7)	0(0.0)	1.000
Discharge time (days), median (range)		1 (1-4)	2 (1-3)	0.957
30-days complications, n (%)		1(6.7)	4 (13.3)	0.651

Results are presented as n (%) or median (range). PSS: Percutaneous Surgical System; LPS: Laparoscopy. BMI: body mass index; EBL: estimated blood loss; LPT: laparotomy.

^a^ Information available for 44 patients.

Furthermore, we evaluated the patients’ post- operative pain using Visual Analog Scale (VAS) at 2h, 4h, 12h, and 24h after surgical procedure and we recorded the cosmetic results and patient satisfaction regarding the size, appearance and healing of scars, before discharge and 30 days after surgery.

### Statistical analysis

Using IBM SPSS Statistics, version 25 for all the analysis, we statistically compared any differences between the PSS-H and LPS-H. Results are represented as percentage for nominal variables and as median (range) for continuous variables. The Shapiro-Wilk test confirmed the abnormal distribution of the continuous variables. For this, the Mann-Whitney-U test for continuous variables and χ2-test or Fisher’s exact test for nominal variables were applied to compare the data of the two groups, as appropriate. Probability (p) values were considered statistically significant for a <0.05 value.

## Results

A total of 45 patients, with BMI ≥ 30, were enrolled, 15 underwent PSS-H and 30 LPS-H. The baseline characteristics are shown in table I. No statistically significant differences were noted between the two groups in terms of age and previous abdominopelvic surgery. Table II gives an overview of perioperative variables. In addition to the total extrafascial hysterectomy, four patients in the PSS-H group and three in LPS-H group received lymph nodal assessment. In all PSS-Hs we performed the procedure using a multifunctional instrument, whereas in LPS-H it was used in 73.3% (p=0.038) of the procedures. Despite this, the OT was similar in the two techniques with a median of 90 mins for PSS-H (range 45-180 mins) and 94 mins for LPS-H (range 22-300) (p=0.587). The closure of the vaginal cuff was performed laparoscopically in 66.7% of the PSS-H cases and in 33.3% of the LPS-H cases, determining a significant difference between the two groups (p=0.034), nevertheless, the vaginal cuff closure time was similar (p=0.094). No differences were found in manipulator usage (p=0.722) as well as in uterine weight, a median of 145 g for the PSS-H group and 180 g for the LPS-H group (p=0.443). The estimated blood loss (EBL) recorded in the PSS-H group (range 50-200 cc) was not statistically different from the EBL recorded in the LPS-H group (range 0-500 cc, p=0.252). Additionally, drain insertion occurred in 53.3% of the PSS-H cases vs 63.3% of the LPS-H procedures (p=0.519). No laparotomic conversions were recorded in either approach. Likewise, no LPS conversions in the PSS-H group were recorded. Only one intraoperative complication (6.7%) was recorded in the control group (EBL ≥ 500 ml), in a 79 year old patient, and no intraoperative complications were recorded in the PSS-H group. Differently, in the PSS-H group, we reported post-operative fever on post-op day 1 (6.7%) in an 80 years old patient affected by endometrial cancer. Discharge time was similar in both groups, with a range of 1-4 day in PSS-H group and 1-3 in LPS-H group, without statistical significance. Within 30 days follow-up time, we recorded one vaginal cuff bleeding in the PSS-H group, whereas for the LPS-H group we reported four (13.3%) 30-days complications; three vaginal cuff bleedings and one vaginal cuff hematoma (p=0.651).

Finally, in Table III, we present the VAS scores for both groups and no differences were recorded. A significant disparity was noted in cosmetic outcome at the discharge (p=0.001), but not at 30 days after surgery.

**Table III:** — VAS Score and Cosmetic Outcome.

VAS Score	PSS		LPS		
Time	median VAS at rest	median VAS after Valsava’s maneuver	median VAS at rest	median VAS after Valsava’s maneuver	P value
2 h	3	4	3	4	0.074 / 0.359
4 h	4	4	4	4	0.073 / 0.901
12 h	4	4	3,5	4	0.654 / 0.950
24 h	4	4	4	4	0.731 / 0.072
					
Cosmetic outcome	PSS	LPS	P value		
median at discharge	9	8	0.001		
median at 30 days	10	9	0.216		

Results are presented as median. PSS: Percutaneous Surgical System; LPS: Laparoscopy. VAS: visual analog scale.

## Discussion

Endoscopic hysterectomy may still be considered a real challenge in obese patients, even for skilled surgeons. The thick abdominal wall increases difficulty during the port placement and limits surgical dexterity. Moreover, intraperitoneal and visceral fat may reduce the visualization of the anatomical structures, raising the risk of vascular, ureteral and nerve injuries. In addition, some studies confirmed an increment of conversion rate to laparotomy and longer OT proportional to an increase of BMI (Walker et al., 2009, Geppert et al., 2011). Nevertheless, the laparoscopic approach seems to be superior to the laparotomic route, in terms of shorter hospital stay, less post-operative pain, earlier return to normal activities, improved quality of life and fewer postoperative complications (Gehrig et al., 2008, Walker et al., 2009).

Consistent with this data and with the continuous developing of technological improvements in MIS in gynecology, there are few studies that investigated the feasibility and the possible limitations of the ultra-MIS techniques in patients with a BMI≥30. Fanfani et al. (2015) analyzed the feasibility of SP technique in obese and non-obese patients, demonstrating that single-site hysterectomy was practicable and safe in obese women. Despite this, the authors showed that the structural differences between SP and LPS may increase the difficulty of the surgical procedure in obese women.

However, to our knowledge, no studies specifically investigated the feasibility and safety of M-LPS in obese patients.

PSS represents the lastest innovation in this field. Even though the feasibility and safety of this approach has been previously described, in the present study, we investigated the influence of obesity in performing a successful percutaneous assisted hysterectomy. Rossitto et al. (2016) reported that the tactile feedback, when performing PSS, could be falsified by the direct contact of the instruments with the abdominal thickness, due to lack of a trocar. As a result, the performance of PSS-H could be restricted in obese women. Despite this, in our study we found that the loss of sensitivity in the PSS technique does not significantly influence the surgical effectiveness resulting in similar results with the standard laparoscopic approach for total endoscopic hysterectomy. Due to the flexibility of percutaneous instruments in manipulation of bulky uteri, described in previous reports (Rossitto et al., 2017, Gueli Alletti et al., 2019), the increase in abdominal thickness could represent an absolute limit in performing a hysterectomy in obese women with bulky uteri.

In our series, although both cases and controls were similar for the most baseline characteristics, the difference reported in BMI data could represent a source of bias.

Previously, some studies comparing standard LPS with other ultra-MIS techniques reported conflicting data in OT (Ghezzi et al., 2011, Park et al., 2014). In contrast, our data showed that PSS-H had similar OTs compared to LPS-H. These differences could be due to the usage of different multifunctional energy devices. In fact, Fagotti et al. (2014), demonstrated that the use of Thunderbeat, used in our PSS-H series, is associated with shorter OT and similar EBL, in comparison to standard electrosurgery (Fagotti et al., 2014).

Although not statistically significant, the differences in the use of drains may be a reflection of the reluctance of the surgeons in the PSS-H group, as the only 5mm port was in suprapubic location, uncomfortable for the patient and not a very useful position.

Over the past few decades, the evolution of MIS has been pushed by the increasing need to offer a better cosmetic outcome to patients, for both benign and malignant gynecologic conditions. In the literature, several manuscripts, comparing different MIS techniques, reported contrasting results about post-operative pain and cosmetic outcome (Yim et al., 2010, Fanfani et al., 2012). Ghezzi et al. (2011), in a randomized trial, demonstrated no significant differences in terms of post-operative pain in M-LPS compared to standard LPS. Our data confirmed no differences in postoperative pain between the two techniques (table III). Interestingly we recorded, a significant difference in cosmetic outcome at discharge but not after 30 days post-surgery. In spite of the small size of provided data, these results may represent the first step to assess PSS as a new frontier in ultra-MIS mainly in the cosmetic outcome, getting closer to the idea of scar-less surgery.

In this context, we can explain our results asserting that, different to SP and M-LPS, characterized by structural limitations in respect to standard LPS, PSS seems to match the accuracy and performance of LPS, maintaining the standard setting and the same instrumental dimensions. SP, using the multichannel trans-umbilical port, requires the usage of instruments with different angulation, limiting the surgical manoeuvres in the abdomen of obese patients. Moreover, this surgical technique is limited to the use of only 2 operative instruments, whereas in a standard LPS-H we can take advantage of additional instruments in separate ports, to use for retraction and dissection (Fanfani et al., 2015). In contrast, M-LPS makes use of the same multiport setting as standard LPS, but the 3-mm instruments, characterized by structural limitations in grasping and manipulation, may be unsuitable to retract voluminous intestinal loops and dissect redundant retroperitoneal fatty tissue (Ghezzi et al., 2011). Differently, PSS, using a multiport setting and 5 mm instruments, seems to simulate the surgical background of standard LPS. Therefore, even if the lack of bipolar instrumentation in percutaneous instruments may be limiting, the usage of a multifunctional energy instrument seems to overcome this potential issue in performing total hysterectomy in obese patients. Undoubtedly, this would represent an additional cost for PSS. Future cost-effectiveness analysis should be performed to estimate and study this aspect of the percutaneous surgical approach in gynecology in this type of patients.

In conclusion, although our data are limited and affected by some inherent bias, we demonstrated for the first time, in a prospective comparison between PSS and LPS approaches, that PSS-H may represent a valid alternative to performing total hysterectomy in obese patients. However, randomized trials are needed to confirm our hypothesis.
